# Recent progress of polymeric microneedle-assisted long-acting transdermal drug delivery

**DOI:** 10.3389/jpps.2024.12434

**Published:** 2024-03-20

**Authors:** Fanda Meng, Xinyu Qiao, Chenglong Xin, Xiaoli Ju, Meilin He

**Affiliations:** ^1^ College of Clinical and Basic Medicine, Shandong First Medical University & Shandong Academy of Medical Sciences, Jinan, China; ^2^ Shandong Center for Disease Control and Prevention, Jinan, China; ^3^ Yantai Key Laboratory of Nanomedicine and Advanced Preparations, Yantai Institute of Materia Medica, Yantai, Shandong, China

**Keywords:** polymeric, microneedles, drug delivery systems, transdermal, long-acting drug release

## Abstract

Microneedle (MN)-assisted drug delivery technology has gained increasing attention over the past two decades. Its advantages of self-management and being minimally invasive could allow this technology to be an alternative to hypodermic needles. MNs can penetrate the stratum corneum and deliver active ingredients to the body through the dermal tissue in a controlled and sustained release. Long-acting polymeric MNs can reduce administration frequency to improve patient compliance and therapeutic outcomes, especially in the management of chronic diseases. In addition, long-acting MNs could avoid gastrointestinal reactions and reduce side effects, which has potential value for clinical application. In this paper, advances in design strategies and applications of long-acting polymeric MNs are reviewed. We also discuss the challenges in scale manufacture and regulations of polymeric MN systems. These two aspects will accelerate the effective clinical translation of MN products.

## Introduction

In recent years, transdermal drug delivery systems (TDDS) have become a popular field for systemic and local drug delivery. Active ingredients are delivered into the circulatory system through dermal tissue to achieve systemic drug administration [1, 2]. Due to the poor permeability of the stratum corneum (SC), the application of TDDS is limited to a few effective and highly lipophilic small molecules (log *p*-values between 1 and 3, with a relatively low molecular weight <500 Da and melting point) [3].

**FIGURE 1:**
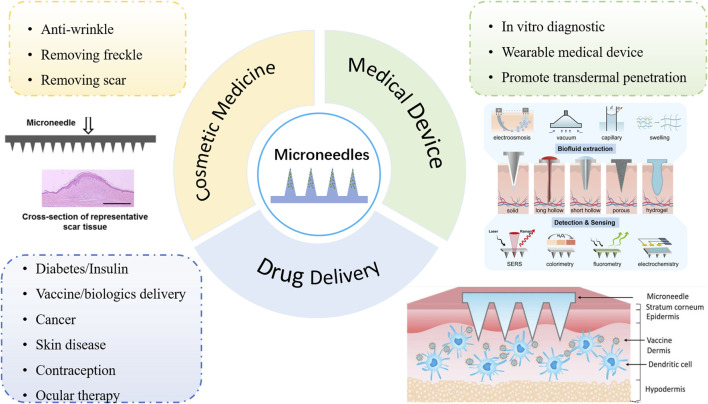
Application fields of MNs: drug delivery, cosmetic medicine, and medical devices. [9] Copyright 2016, Materials Science and Engineering: R: Reports. [10] Copyright 2023, Biomaterials science. [11] Copyright 2022, Journal of Controlled Release.

MNs are typically composed of multiple micron-sized humps, ranging in size from 25 to 1,000 microns, assembled on the bottom layer of the supporting substrate or one side of the patch [4]. As a physical permeation-enhancing technique, it can penetrate the stratum corneum and form reversible microchannels on the surface layer of the skin, greatly improving the permeability and penetration ability of drugs [5–7]. This can avoid the invasiveness of conventional injection administration and improve acceptance and compliance in patients. Six major categories of MNs have been developed since the concept was first proposed in 1976: solid, coating, hollow, swell, dissolving, and biodegradable [8]. In recent years, the application field of MNs has gradually expanded to drug delivery, cosmetic medicine, and medical devices. Specific applications in each field are shown in Figure 1. The materials for preparing MNs are divided into inorganic materials and polymers. The inorganic materials are, typically, silicon and metal, which are used to make solid or hollow MNs [12]. However, these inorganic materials often have low biocompatibility and are prone to fracture, resulting in intradermal biohazard residue. Biodegradable polymers, meanwhile, have gradually become the preferred materials for MNs preparation due to their good mechanical properties, excellent biocompatibility, and low preparation cost. This kind of MN can be safely degraded *in vivo*, even if breakage in skin tissues occurs, without any sharp biohazardous waste.

In recent years, with the development of slow-releasing polymer and nano/micro-particles drug delivery technology, MNs have evolved from the classical rapid release mechanism to the long-acting release mechanism [13]. Currently, a wide range of drugs can be delivered via MNs, including chemical drugs and macromolecular biological drugs such as proteins and vaccines. Patients with chronic diseases require long-term medication and have poor compliance. Traditional rapid-release delivery systems (e.g., daily oral or injections) cause the daily plasma concentration of the therapeutics to fluctuate greatly, which increases the toxicity risk. In addition, delayed and missed medication could lead to ineffective control of the disease, such as blood glucose control for diabetics and contraception for women of childbearing age. Long-acting delivery systems can maintain a gentle plasma drug concentration, thereby avoiding higher systemic toxic exposure. Therefore, long-acting MNs are expected to improve self-administration management and prolong therapeutic efficacy in patients with chronic diseases [14]. Polymeric MNs can achieve long-acting drug release by changing the degradation curve of the polymer excipients and the diffusion curve of the encapsulated drug [15–17]. In this review, we focused more on the advantages of polymeric MNs in the treatment of chronic diseases. We summarized four representative design strategies for long-acting MNs, and then discussed the action mode of long-acting MNs in different disease areas. We concluded by discussing the challenges of this technology and the possibilities of moving from the laboratory to the clinic in the future.

## Design strategies of long-acting polymeric microneedles

**FIGURE 2:**
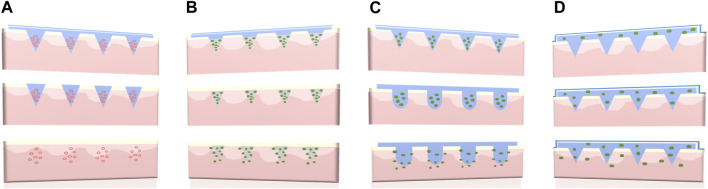
Four different types of long-acting polymeric MNs. **(A)** Nano/microparticle-loaded dissolving MNs, **(B)** Biodegradable polymeric MNs, **(C)** Swellable polymeric MNs, and **(D)** Back-layer reservoir polymeric MNs.

Long-acting polymeric MNs have an extended medical effect by constantly releasing drugs over a prolonged period of time [18, 19]. According to the characteristics of the main polymer materials (dissolving, biodegradable, or swelling) and the types of drug reservoir (nano/microparticles or back-layer), four representative design strategies of long-acting polymeric MNs could be summarized as follows: (A) Nano/microparticle-loaded dissolving MNs, (B) Biodegradable polymeric MNs, (C) Swellable polymeric MNs, and (D) Back-layer reservoir polymeric MNs. The schematic diagram is shown in Figure 2. In addition, different design strategies of long-acting polymeric MNs along with advantages and limitations are shown in Table 1.

**TABLE 1:** Different design strategies of long-acting polymeric MNs along with advantages and limitations.

Design strategies	Mechanism of action	Advantages	Limitations
Nano/microparticle-loaded dissolving MNs	Poke, dissolve, and release	Both hydrophobic and hydrophilic drug	Combined with nanocarrier technology, the process is complicated
		The matrix excipients are soluble	The drug loading is limited
		The application time is short	
Biodegradable polymeric MNs	Poke, separate, deposit, and release	The release curve can be regulated according to the physical and chemical properties of the polymer	The drug loading capacity is limited
		Multilayer design could shorten the application time	A fast separation structure needs to be designed
Swellable polymeric MNs	Poke, swell, and release	MNs baseplate could also act as a drug reservoir	It is mainly used in hydrophilic drugs and biological macromolecules
		Almost no polymer remains in the skin after removal	The type of preparation material is limited
			It should be always applied to the skin during administration
Back-layer reservoir polymeric MNs	Poke, diffuse, and release	It could meet the requirements of sufficient drug loading	The microchannels formed by the dissolving needle-tips are easy to close and prevent the drug from diffusing continuously
			It should be always applied to the skin during administration

### Nano/microparticle-loaded dissolving microneedles

The matrix excipients of nano/microparticle-loaded dissolving MNs are usually water-soluble polymer materials such as hyaluronic acid (HA), polyvinylpyrrolidone (PVP), polyvinyl alcohol (PVA), chondroitin sulfate (CS), glucan, and so on. Slow-release systems such as microspheres and nanoparticles are used to envelop drugs in the soluble matrix of needle-tips [20, 21]. Generally, such slow-release nano/microparticles can be composed of liposomes, metal nanoparticles, nanocrystals, etc., which can play a sustained release role when combined with MN delivery systems [13]. The matrix excipients of this kind of MN would fully dissolve within several seconds or minutes after being inserted into the skin, leaving microspheres or nanoparticle deposits into the skin to act as a drug reservoir [22–24].

Ito et al. encapsulated porous silicate microparticles absorbing insulin in long-acting dissolving MNs using chondroitin sulfate as a base material [25]. They evaluated the hypoglycaemic effect of the MNs in mice. It showed that blood glucose levels started to decrease after the administration of MNs loaded with insulin-absorbed porous silicate particles. The minimum glucose levels were observed at about 2 h and the hypoglycaemic effect was sustained until 8 h. In another study, Wu et al. prepared ovalbumin-encapsulated polylactic acid co-glycolic acid (PLGA) nanoparticles using the W/O/W double emulsification method and then loaded the nanoparticles into the needle-tips of the bilayer-dissolving MNs [26]. The model protein drug was sustainably delivered to the ocular scleral tissue in a minimally invasive manner for 2 months. This approach significantly improved patients’ compliance and provided an effective alternative for the treatment of chronic scleral diseases. In the future, potent anti-VEGF biotherapeutics could be more cost-effective via this bilayer MN platform. Similarly, Zhang et al. developed biodegradable MNs that contained tetracycline-loaded PLGA nanoparticles and cytokine-loaded silica microparticles for sustained release into local gingival tissue to achieve the regeneration of periodontal tissue in periodontal disease [27]. Tekko et al. loaded the poorly water-soluble methotrexate nanocrystals into PVP/PVA dissolving MNs for intradermal delivery. *In vivo* studies of Sprague-Dawley rats showed that methotrexate could be delivered locally to the skin for 72 h, reducing systemic exposure in order to avoid side effects and enhance treatment of psoriasis [28]. Peng et al. prepared the dissolving MNs by pulverizing antifungal drug amphotericin B with PVA and PVP to form micronized particle-loaded gels. This simple preparation of microparticle-loaded MNs localized amphotericin B inside the skin for a week for treatment of cutaneous fungal infections [29]. Moreover, the combination of microparticles such as liposomes with MN delivery systems also greatly improves bioavailability and particle stability in targeted delivery of drugs for the treatment of alopecia [30].

### Biodegradable polymeric microneedles

Biodegradable polymers have attracted a great deal of attention due to their bioavailability and ability to precisely control the degradation rate of drug delivery systems. These polymers are used in the delivery of antibiotics, growth hormones, and vaccines in the form of microcapsules, films, fibres, and so on [31, 32]. Biodegradable polymeric MNs resemble a combination of MNs and implantable drug deposits [7]. Polymers for this kind of MN are classified into natural polymers (such as chitosan or silk fibroin) [33, 34] and synthetic polymers [such as PLGA and polylactic acid (PLA)] [35, 36], which are almost insoluble in the interstitial fluid of skin tissue but could degrade slowly. The drug release curve is closely related to polymer degradation rate. Thus, the physical and chemical properties of the polymer could be modified (for example, molecular weight and molar ratio of the blocks) to adapt to the release period of different drug types [7, 37]. In addition, it is preferable to manufacture such MNs using double-layer or multilayer techniques, so that the biodegradable cone needle-tips can be quickly separated from the back layer-base once applied to the skin to shorten the application time [28, 38, 39].

**FIGURE 3:**
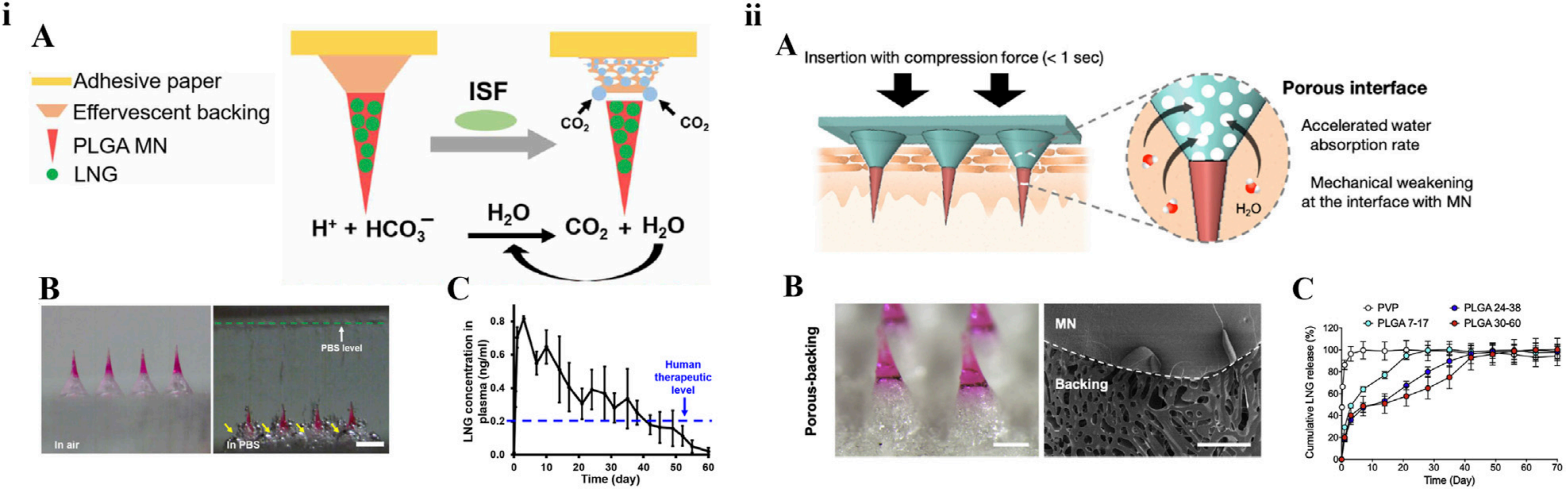
Design strategies of a rapidly separable degradable polymer MN patch for contraception. **(i)** Effervescent MN patch: A) Schematic plot. B) Microscope images before and after MN patch was placed in PBS solution. C) Pharmacokinetics in rats [42] copyright 2019, Science Advances. **(ii)** Porous-backing MN patch: A) Schematic plot. B) Microscopy and scanning electron microscopy images. C) *In vitro* release curve [45] copyright 2021, Journal of Controlled Release.

Chitosan is often used as a material for biodegradable polymeric MNs due to its good biodegradability. The MNs patch prepared by Chen et al. consisted of chitosan needle-tips loaded with H1N1 influenza vaccine and a dissolvable supporting array [40]. After insertion into the skin, the supporting array could quickly dissolve upon contacting the interstitial fluid of the skin and the chitosan needle-tips were entirely implanted into the dermis tissue to achieve continuous intradermal delivery of influenza vaccine for at least 16 weeks. By providing sustained antigen exposure and immune stimulation, the immunogenicity of the inactivated influenza vaccine was enhanced, and long-lasting protective immunity was stimulated in mice. Similarly, Castilla-Casadiego et al. tested biodegradable chitosan MN patches loaded with meloxicam. The results showed that the release of meloxicam *in vitro* for 7 days was about 33.02 ± 3.88% and the sterilization process of ethylene oxide had no effect on the morphology and composition of MNs. This chitosan-degradable MN had a positive effect on pain management in cattle [41]. PLGA is also often used for sustained drug release due to its special intrinsic properties, including biocompatibility and biodegradability, which provide good applicability for the preparation of MNs. In 2019, Prausnitz’s group reported a rapidly separable degradable polymer MN patch with PLGA needle-tips for sustained-release of the contraceptive hormone levonorgestrel (LNG) [42]. With the effervescent contact between the back-layer and interstitial fluid of skin, degradable needle-tips would remain embedded into the skin after being pressed against the skin for 1 min. According to the pharmacokinetics in rats, the effervescent MN patch could keep the plasma concentration of LNG above the human contraception level for more than 1 month. This study was the first to use the effervescent technique in MNs. The biodegradable MNs prepared by Abu-Much et al. for intradermal delivery had the PLGA needle-tips loaded with furosemide and a flexible support layer composed of sodium gluconate and glycerol. This study overcame the limitations of frequent hospitalizations for intravenous drip and improved compliance in patients with congestive heart failure [43]. Afterwards, Yang et al reported a sustained-release MN patch loaded with growth hormone. This report also used the effervescent strategy to actively separate silk needle-tips to provide the delivery of growth hormone for up to 7 days [44]. In 2021, Prausnitz’s group reported another immediate MN detachment system. MNs were strong enough to penetrate the skin but enabled immediate detachment within 1 s because of the porous patch backing [45]. Their efforts focused on acceptable and simple attractive long-acting contraception methods. The design strategies of a rapidly separable degradable polymer MN patch for LNG are shown in Figure 3.

### Swellable polymeric microneedles

Swellable polymeric MNs are usually prepared by crosslinking hydrophilic polymers. Polymer cross-linking methods generally include chemical cross-linking and physical incubation under UV radiation or high temperature [46]. The materials prepared for the swellable polymeric MNs do not dissolve or degrade in the skin tissue. Instead, by absorbing the skin interstitial fluid, the needle-tips of these MNs would swell to create hydrogel channels for drug diffusion. Therefore, the swellable MNs should be always applied to the skin during administration. Drug release kinetics were mainly affected by drug solubility, the crosslinking degree and swelling properties of the polymer, and polymer-drug interaction [47]. Usually, the swellable MNs are designed as an integrated type, so that the baseplate can also be used as a drug reservoir. Therefore, it is possible to deliver milligrams of drug on small areas [48]. In addition, this type of MN can be completely removed after application, eliminating the safety problem of polymer and degradation product residues [49].

Yang et al. invented new phase-transition hydrogel MNs by taking advantage of the unique PVA-forming microcrystalline domains as cross-linking junctions [50]. PVA microcrystalline crosslinking was processed via mild freeze-thaw cycle treatment. Due to the absence of chemical and ionic cross-linking agents, the swellable MNs were friendly to protein drugs. In this research, they encapsulated insulin in the needle-tips to achieve sustained release for blood glucose control. Shi et al. designed a PVA-swellable MN patch loaded with chitosan lactate and exosomes for painless hair-loss treatment. The continuous release of chitosan lactate and exosomes synergistically promotes hair regeneration by regulating hair follicles [51]. In another study, Yang et al. reported swellable MNs based on acrylic resin (EUDRAGIT RL100), which carried up to 2.1 mg of the hydrophobic drug granisetron base (GRB) on 1 cm^2^/piece. A pharmacokinetics study in rats has shown that controlled delivery of GRB was successful for more than 7 days by adding the pore-forming agent CaHPO_4_ and PVP [52]. In addition, Zhao et al. designed MNs with a swellable gelatin methacryloyl (GelMA) needle body and a dissolvable PVA backing layer in 2022 [53]. Methacrylic anhydride interacted with gelatin at 50°C to create GelMA and formed a cross-linked network in the presence of UV light. The cross-linked GelMA needle body showed strong swelling properties and significant slow release of donepezil. In addition, some studies have designed swellable MNs to extract interstitial fluid (ISF) and determine real-time pH so as to build a detection platform with sensors [54]. Previous studies have shown that 2-ethoxyethanol (ECS)-modified silk fibroin swelling MNs can easily penetrate porcine skin to a depth of ∼200 μm *in vitro* and transform into semi-solid hydrogels with a porous network of 50–700 nm inside for higher transdermal release kinetics [55]. All these above studies reveal that swellable polymeric MNs can take advantage of the skin swelling properties to extract ISF and deliver drugs; they also offer great benefits in improving patient compliance.

### Back-layer reservoir polymeric microneedles

For back-layer reservoir polymeric MNs, the needle-tips are used as a tool to create drug penetration channels after application to the skin. The size of needle-tips may be too limited to meet the requirements of sufficient drug loading for long-term administration. Therefore, it is necessary for external drug reservoirs or the MN base layer as drug reservoirs to improve the drug loading capacity of MNs for long-acting drug delivery [7, 56, 57]. The materials used to prepare the needle-tips are usually dissolving or swellable polymers [48, 58]. As previously mentioned, the swellable needle-tips can prevent the closure of the skin microchannels to achieve continuous drug delivery from reservoirs.

In 2008, it was reported that polysaccharide MNs backing-layer as a drug reservoir was feasible for sustained drug release, for example sulforhodamine [59]. After this discovery, the backing-layer of MNs were widely used as drug reservoirs for long-acting drug delivery. Zhu et al. reported an insulin-loaded composite MN patch. The needle-tips were prepared with silk fibroin that could quickly dissolve in the skin and promote insulin release. At the same time, the swellable but not dissolvable base-layer acted as the reservoir of insulin to achieve sustained release through the microporous channels created by needle-tips [60]. Moreover, Chen et al. also designed PVA-coated MNs for efficient skin penetration and glucose-tolerant insulin sustained delivery. The needle-tips coated a phenylboric acid smart gel onto a PVA gel layer that acted as the reservoir for insulin and supported MNs puncture capabilities [61]. Migdadi et al. reported that a combination hydrogel MN patch could deliver high doses of metformin hydrochloride continuously for 24 h via the skin. The composite MNs patch consisted of two layers. The swellable MNs layer was made of polyethylene glycol (PMVE/MA Gantrez^®^ S-97) crosslinked with polyethylene glycol (PEG), and the drug reservoir base-layer was attached by a lyophilized patch loaded with metformin hydrochloride. Drug-free needle-tips swelled to form hydrogels by absorbing skin interstitial fluid, thus providing continuous and non-blocking channels from the backing reservoir to the skin microcirculation [62]. In 2023, it was reported that hydrogel MNs were integrated with a PEG reservoir to facilitate the transdermal delivery of sildenafil citrate [63]. Courtenay et al evaluated the hydrogel MNs with polymer films and freeze-dried layers containing esketamine as drug reservoirs for drug delivery in treatment-resistant depression. Reservoir candidate MNs could achieve esketamine plasma concentrations higher than the therapeutic levels of 0.15–0.3 μg/mL over 24 h in female Sprague-Dawley rats [64].

In addition to small molecule drugs, Courtenay et al. also reported a hydrogel combination MN patch for biologic treatment of the large molecule drug bevacizumab. This work is the first to report high-dose delivery of antibody therapies based on a microneedle platform, while highlighting the potential to provide continuous delivery to lymphatic and systemic circulation [65].

## Application of long-acting polymeric microneedles

Long-acting formulations should maintain drugs within therapeutic levels and deliver them for as long as treatment is needed. Many drugs, such as biomacromolecules, drugs with low bioavailability or long half-life, and drugs delivered locally, are ideal candidates for long-acting polymeric MNs [3]. The development of long-acting MNs for controlled drug release can significantly improve the therapeutic effect, reduce the frequency of administration, prevent potential toxicity caused by systemic exposure, and improve poor medication compliance in patients with chronic diseases. The following sections introduce their application in diabetes, vaccine, cancer, skin diseases, and contraception.

In Table 2, we summarize the long-lasting polymeric MNs design strategies and their application areas mentioned in this paper.

**TABLE 2:** Different design types and application areas of long-acting polymeric MNs.

Design strategy	Drug	Application	Materials of MNs	Release duration	Reference
Nano/microparticles dissolving MNs	Insulin	Diabetes	Porous silicate adsorbents, chondroitin sulfate	8 h	[25]
	Tenofovir	HIV	PLGA	5 days	[66]
	Finasteride	Benign prostatic hyperplasia, Androgenic alopecia	PLGA	14 days	[67]
	Ovalbumin (OVA)	Eye disease	PLGA, PVA, and PVP	2 months	[26]
	Methotrexate	Psoriasis	PVA and PVP	3 days	[28]
	Insulin	Diabetes	Carboxymethyl cellulose	12 h	[68]
	OVA and rHBsAg	Vaccine	PLGA, PVA	2 months	[69]
	Etonogestrel	Contraception	PVA and HPMC	1 week	[70]
	Anti-PD-1 and glucose oxidase	Cancer	HA and pH-sensitive dextran nanoparticles	10 days	[71]
Biodegradable polymeric MNs	H1N1 influenza vaccine	Influenza	Chitosan	16 weeks	[40]
	Levonorgestrel	Contraception	PLGA/PLA	1–4 months	[42, 72–74]
	Growth hormone	Protein hormone	Silk, polyacrylic acid	7 days	[44]
	Three types of insulin	Diabetes	PVA, PLA, Gel and HA	12 h	[75]
	Doxorubicin	Cancer	Gelatin methacryloyl	24 h	[76]
	Tumor lysates and melanin	Cancer	Cross-linked HA, N,N’-methylenebisacrylamide	5 days	[77]
	Natural polyphenols	Atopic dermatitis	HA, PLGA, PVA and PVP	2 months	[78]
	Levonorgestrel	Contraception	PLLA, PLA, PLGA, PVA and sucrose	6 months	[74]
	Etonogestrel	Contraception	PLGA	2 weeks	[79]
	DNA vaccines	vaccine	poly (β-amino-ester)	1 week	[80]
	OVA	food allergy	Silk fibroin	2 weeks	[81]
Swellable polymeric MNs	Insulin	Diabetes	PVA	6 h	[50]
	Granisetron base	Antiemetic	Acrylic resin (EUDRAGIT RL100)	7 days	[52]
	Insulin	Diabetes	Silk fibroin protein and phenylboric acid/acrylamide hydroge	Smart delivery (autonomously on the demand)	[82]
	Insulin	Diabetes	polystyrene-block-poly (acrylic acid)	12 h	[83]
Back-layer reservoir polymeric MNs	Insulin	Diabetes	Silk fibroin	8 h	[60]
	Metformin hydrochloride	Diabetes	PMVE/MA Gantrez^®^ S-97	24 h	[62]
	Bevacizumab	Chemotherapeutic	PVA	7 days	[65]
	Methotrexate	Psoriasis	PVA and PVP	24 h	[84]
	Recombinant human growth hormone	Growth hormone deficiency	Silk protein	7 days	[85]

### Diabetes

**FIGURE 4:**
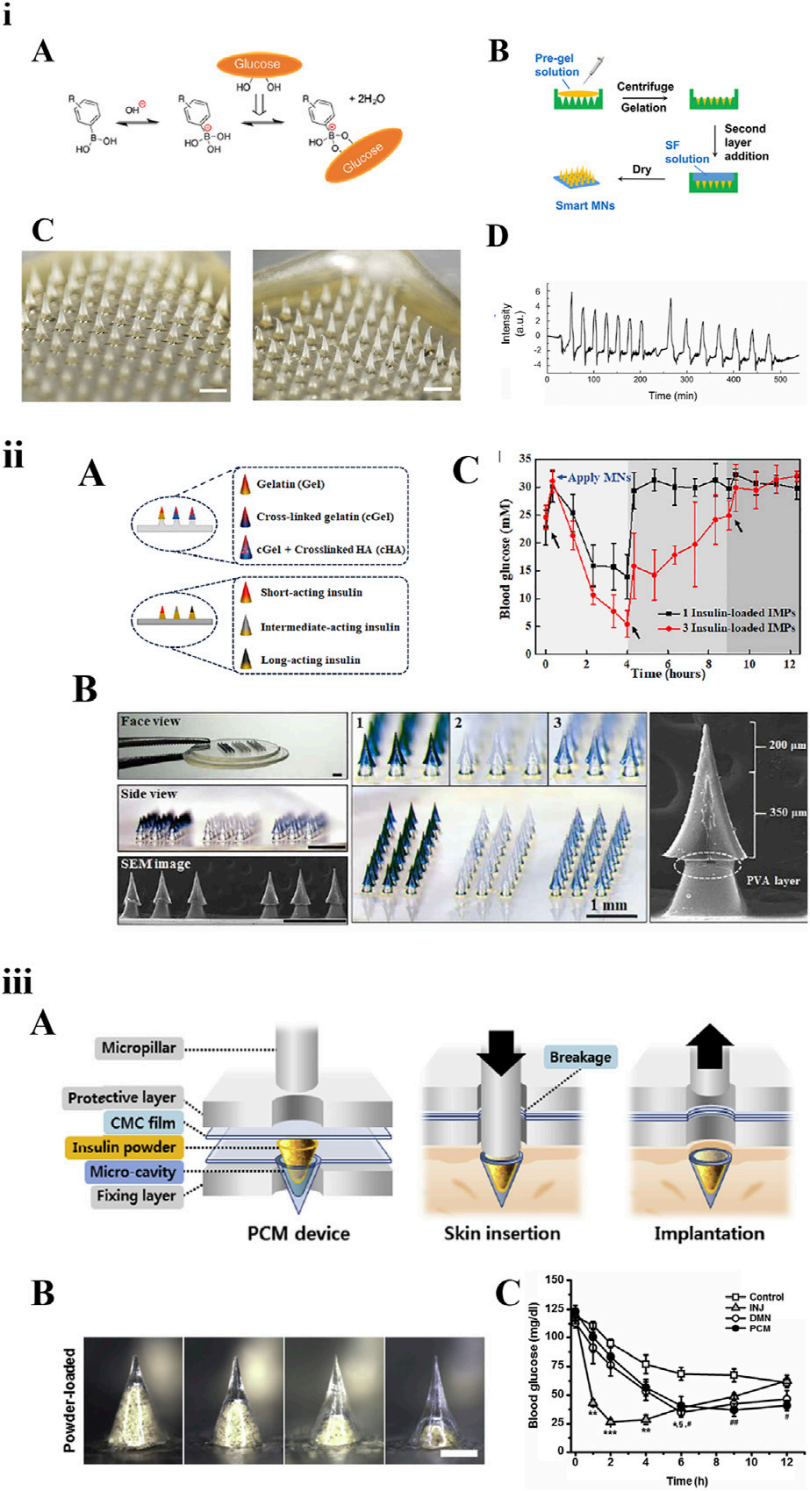
Three types of long-acting polymeric MNs loaded with insulin. **(i)** Smart MNs fabricated with a two-layer strategy: A) The equilibria between phenylboronic acid (PBA) derivatives and glucose. B) Fabricating schematic. C) Morphology of MNs before and after being inserted into skin within 3 h. D) *In vitro* insulin release in response to glucose [82] copyright 2019, American Chemical Society. **(ii)** Basal-bolus insulin-integrated MN patch: A) Schematic of MNs fabricated by three types of materials or loaded with three types of insulin. B) Microscope images of the integrated MNs. C) Blood glucose levels of diabetic rats after application of integrated MNs [75] copyright 2020, Science Advances. **(iii)** MNs loaded with insulin powder: A) Fabrication and application schematic. B) Microscope images of a single micro-cavity loaded with drug powder. C) Blood glucose levels [68] copyright 2020, Biomaterials.

Diabetes is a common chronic disease associated with blood glucose disorder. Frequent subcutaneous injections of insulin are the most common treatment to effectively control blood glucose levels. For insulin delivery based on MNs, medical waste can be reduced and problems such as injection pain, needle fear, and adverse reactions at the injection site can also be solved. Three types of long-acting insulin MNs are shown in Figure 4.

The smart glucose-responsive MN patches are a promising diabetes management strategy for insulin delivery systems. Chen et al. reported a smart insulin MN based on silk fibroin binding semi-interpenetrating network hydrogels. The preparation method of the MNs was optimized into a two-layer strategy: the needle-tip layer was composed of silk fibroin protein and phenylboric acid/acrylamide hydrogel, and the base layer was prepared with only silk fibroin. This stimulatory response system was based on the reversible binding reaction of glucose with borate in aqueous solution [82]. Therefore, MNs could accurately function like a healthy pancreas in response to hyperglycaemia to release insulin autonomously on demand. Dual-responsive MNs, which are dependent on glucose and H_2_O_2_ concentration and regulate the rate of insulin release, have been used to load insulin as early as 2018 [86]. In the future, through integration with electronic messaging technology, wearable insulin-MN patches would be more attractive.

Chen et al. developed an integrated MNs patch containing three different compartments using two strategies. The three regulating compartments either contained three types of insulin (short-acting, medium-acting, and long-acting) or were fabricated by combining three materials. *In vivo* studies have shown that three types of insulin could achieve regulated delivery by different polymeric materials to improve postprandial blood glucose fluctuations [75]. Kim et al. reported one type of high-dose MN patch carrying insulin powder. Insulin powder in the micro-cavity was pressed into the skin using a micropillar implantation system. Compared with subcutaneous injection, it could not only overcome the loading capacity and activity loss, but also showed long-lasting hypoglycaemic effect [68]. Seong et al. proposed a new method for insulin transdermal delivery using bullet-shaped double-layered MNs with water-swellable tips. The bullet-shaped needle-tips could interlock with the soft tissue of skin after swelling, significantly improving adhesion strength. It could prolong insulin release by passive diffusion, help regulate blood glucose levels, and improve the delivery platform for a variety of protein drugs that require sustained release kinetics [83]. In addition, several studies have been designed based on swellable and reservoir MNs to provide long-lasting release of intradermal insulin [87].

Previous studies have also shown that insulin encapsulated in polymeric MNs could maintain its biological activity and did not require cold chain transportation and storage, which was convenient for patients to use [88, 89]. However, long-term studies on the cytotoxicity and biocompatibility of insulin by the MNs route of delivery are lacking. When promoting the application of MNs, the sterility and bacterial endotoxin should be carefully checked to avoid clinical infection [90]. Moreover, the frequency of MNs administration should be evaluated carefully for diabetic patients, given the small volume of the moulds and the small amount of insulin loading [91].

### Vaccine

Over the past 20 years, MN patches have contributed to great medical advances in the systemic delivery of vaccines and biologics. Compared with muscle and subcutaneous space, the intradermal antigen-presenting cells are abundant and effective and can produce stronger immune responses at low doses [92]. MNs are the strategy for intradermal vaccine administration [93], and can also overcome adverse events of injection administration, such as pain, inflammation, and abscess reactions [6, 94]. The long-acting vaccine MN delivery system can also prolong antigen presentation time, thereby inducing durable immunity and producing a stronger immune response [95].

**FIGURE 5:**
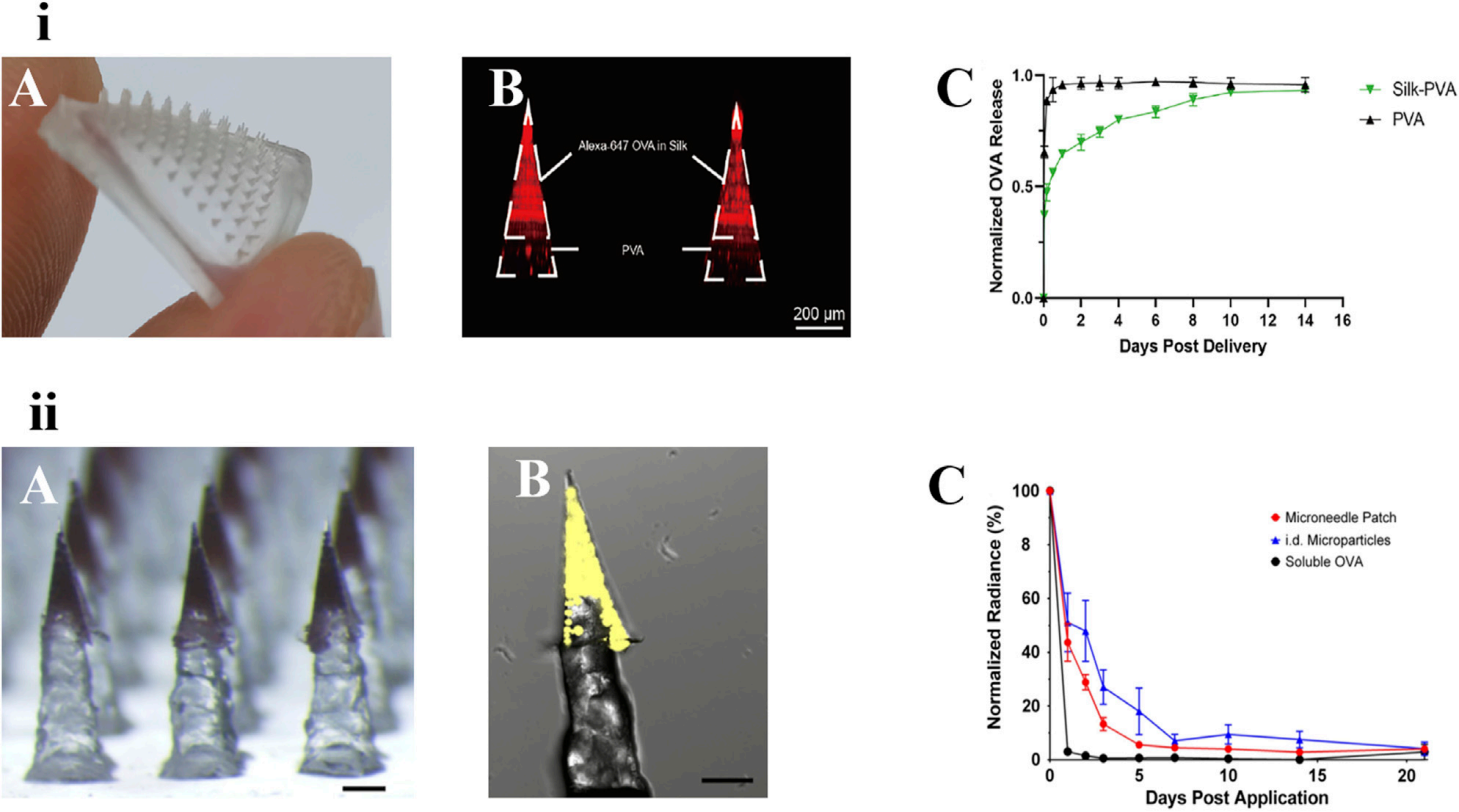
Two types of long-acting polymeric MNs for vaccine. **(i)** An implanted silk-PVA composite MN patch: A) Optical image of the MN patch. B) confocal image of this MN patch showing Alexa-647 OVA (red) was restricted in needle-tips. C) Release kinetics of OVA loaded in the composite MN patches or PVA MN patches [81]. copyright 2022, Journal of Controlled Release. **(ii)** Vaccine MNs containing PLGA controlled-release particles: A) Pedestal patch with sulforhodamine B. B) Confocal image of individual pedestal needle containing microparticles loaded with Ovalbumin-Alexa Fluor 647 (fOVA) conjugate. Scale = 250 μm. C) fOVA-loaded ASE microparticles remain in the skin for several days [97]. copyright 2018, Wiley.

DeMuth et al. reported the use of MNs coated with a releasable polyelectrolyte multilayer film. The polymer films carrying DNA, immune-stimulating RNA, and biodegradable polycations could be rapidly implanted into immune cell-rich epidermis after short administration of MNs. Local transfection of DNA was enhanced and allowed to persist in the skin for days to weeks, with release kinetics determined by film composition. Pharmacodynamics showed that this strategy could induce immune response to model HIV antigens in mice and enhanced the production of memory T cells. In nonhuman primate skin, this DNA MN elicited 140-fold higher gene expression than intradermal injection [96]. Zhang et al. proposed a novel implantable MN for specific food allergy immunotherapy. The MNs, which consisted of ovalbumin (OVA)-loaded silk fibroin needle-tips and a flexible PVA pedestal, induced a long-acting immune response for at least 2 weeks [81]. Two types of long-acting vaccine MNs are shown in Figure 5.

The reliance on repeated hypodermic injections for vaccines is one of the biggest barriers to increasing global vaccination coverage and protection. The MNs delivery method using nano/microparticle-encapsulated vaccines provides a novel method of different vaccine delivery systems for skin immunization [81]. Mazzara et al reported vaccine MNs containing PLGA controlled-release particles to achieve the same performance as traditional prime-boost immunity of conventional hypodermic injections. A variety of antigens [e.g., OVA and recombinant hepatitis B surface antigen (rHBsAg)] were encapsulated in PLGA particles containing trehalose and Alhydrogel adjuvants through aqueous active self-healing encapsulation technique. The particles were then incorporated into polymeric pedestal-based MNs and deposited intradermally with the MNs dissolved rapidly for intradermal vaccination. *In vitro*, the microparticles exhibited a biphasic release characteristic, with soluble antigen burst release followed by a delayed release of the Alhydrogel-antigen complex for approximately 2 months. This new MN platform could provide a foundation for patients to self-administer vaccines [69].

### Cancer

Traditional cancer treatments, including surgery, chemotherapy, and radiotherapy, can damage not only tumor cells but also healthy tissues [98]. MN drug delivery has shown special advantages in the treatment of superficial tumours, and its minimally invasive treatment features are more attractive than invasive drug delivery methods (such as intravenous infusion) [3, 99]. Long-acting MNs can directly accumulate drugs into superficial tumor lesions and achieve controlled release, reducing systemic toxicity. Its sustained-release properties can also reduce repeated medical visits, increase medication compliance, and reduce the possibility of tumor recurrence [100–102].

Luo et al. prepared a biodegradable MN patch containing the chemotherapeutic drug doxorubicin (DOX) with a one-step forming technique. The mechanical properties and drug delivery rate of this MN patch could be adjusted according to the UV photocuring crosslinking degree of the GelMA. GelMA MNs crosslinked for 60 s showed only 20% degradation and released DOX for 24 h. This study demonstrated the anticancer effect of this GelMA MN patch on melanoma cell line A375 [76]. In recent years, great achievements have been made in cancer therapy for immunotherapy by the immune system, including cancer vaccines and checkpoint inhibitors. These treatment methods are designed to reshape the tumor microenvironment, generate the system immune response, and ultimately trigger the effective CD8^+^ T cell response to clear cancer cells [103, 104]. However, the great challenge hindering the clinical translation of immunotherapy is the need to avoid systemic toxicity [105, 106]. Thus, MNs, which can reach superficial lesions and have sustained-release properties, provide a good platform for anticancer therapeutic methods. Wang et al. developed a self-degradable MN patch consisting of pH-sensitive dextran nanoparticles loaded with anti-PD-1 and glucose oxidase. This MN patch could sustain the release of anti-PD-1 for potential treatment of melanoma. After insertion into the skin, with the oxidation of glucose and the production of H^+^, the pH was reduced, which triggered the degradation of the nanoparticles, resulting in the continuous release of anti-PD-1. Compared with ordinary non-self-degradable MNs of free anti-PD-1, this self-degradable MN could induce stronger immune response and anti-tumor efficacy [71]. Zeng et al. designed a MN delivery system based on a dopamine-structured nanoshell to coat glucose oxidase, which can increase tumor starvation and inhibit tumor growth with few side effects [107]. Ye et al. loaded B16F10 whole tumor lysate and photothermal reagent into MNs prepared by crosslinked methacrylate hyaluronic acid. The results showed that the developed MNs could continuously release loaded tumor lysates and enhance antigen uptake by dendritic cells under laser exposure. This photothermal response enhanced T cell migration and local proinflammatory cytokine production and effectively targeted both primary and distal secondary tumours [108].

### Skin diseases

Skin is an important protective barrier for the human body. Chronic skin diseases seriously affect people’s life quality [109]. In order to reduce systemic side effects, topical therapy is mainly recommended for mild cases. But the barrier nature of the skin stratum corneum reduces the therapeutic efficacy of this method. Researches have shown that, when traditional cream was applied to the skin surface, at most 20% of the drug could be absorbed into skin [110]. In combination with long-acting preparations, polymeric MNs have shown significant advantages due to their safe and efficient intradermal delivery of drugs. To date, polymeric MNs have been widely studied to be used to treat skin diseases such as hypertrophic scars, acne, actinic keratosis, and atopic dermatitis [111–115]. For the application of MN-based devices in the treatment of skin diseases, the design principles for improving efficacy are grouped into the following four points: i) enhancing skin penetration, ii) controlled drug release, iii) Targeted drug delivery, and iv) imaging and therapeutic functions [116].

Psoriasis is a chronic inflammatory autoimmune skin disease which requires long-term treatment and affects 1–3% of the population. Tekko et al. prepared a novel transdermal patch for methotrexate delivery that integrated PVA/PVP hydrogel-forming MNs and a methotrexate reservoir-patch. The preclinical studies in rats showed that this integrated MN patch could deliver methotrexate transdermally over 24 h in a sustained manner, effectively reducing the peak concentration of drugs in blood compared with the oral methotrexate aqueous solution group [84]. Thus, the novel transdermal delivery system had potential to avoid a gastrointestinal reaction or reduce side effects associated with high peak blood concentration, such as nausea and vomiting, to address the barriers to long-term treatment of skin diseases. Cyclosporine A (CyA) has a large molecular size (1202Da) and is hydrophobic. Jeong et al. developed dissolving MNs based on the co-solubility of CyA and hydroxypropyl cellulose for local and sustained intradermal administration. In a rat model, the dissolving MNs performed well *in vivo* and could maintain plasma CyA concentrations above 5 ng/mL for 72 h, but oral administration was only sustainable for 24 h. Due to its hydrophobicity and high molecular weight, CyA could remain in the skin for a longer time and be absorbed slowly into the body. The use of dissolved MNs would improve the safety of CyA delivery and achieve high bioavailability [117]. Atopic dermatitis is a chronic inflammatory skin disease that requires long-term treatment to effectively control the symptoms [118]. Chen et al reported a double-layered PLGA/HA MNs for sustained delivery of polyphenols, curcumin (CUR), and gallic acid (GA) [78]. When applied, the HA layer could quickly dissolve and release GA, while the PLGA tips-layer could provide sustained release of CUR for 2 months. In the initial stage of administration, GA and CUR acted synergistically, and CUR maintained the curative effect in the later stage. This study provided a dual treatment approach for atopic dermatitis, both fast- and long-acting. MNs combined with nanocarriers can also be used to target hair follicles to deliver drugs for the treatment of local skin diseases such as alopecia and acne [116, 119].

### Contraception

The high rate of unintended pregnancy creates serious economic and emotional burdens for society and women. Long-acting contraceptives can improve family planning and provide women of childbearing age with flexible protection. Long-acting contraceptives on the market include long-acting injections, implants, and the vaginal ring. But they all are invasive and need to be administered or removed by healthcare professionals [120]. Therefore, there is an urgent need for improved self-administered long-acting contraceptives to expand choice and provide convenience for women. Although the transdermal patch provides up to 1 week of contraceptive protection, it is conspicuous and interferes in daily hygiene because of continuous application [121]. Long-acting polymeric MNs could address these shortcomings.

In the section “*Biodegradable Polymeric microneedles*” of this paper, the rapidly separable biodegradable polymeric MNs patch reported by Prausnitz’s group was introduced for the intradermal delivery of LNG [42, 45]. Recently, they have reported one contraception MN patch with a core-shell structure that provides LNG zero-order release for more than 6 months *in vitro* [74]. He et al. reported a long-acting dissolving MN loaded with the third surrogate hormone etonogestrel (ENG) microcrystal particles [70]. PVA was used to make the flexible back-layer of MNs so that it was more tightly attached to the skin. When the MNs were applied on the skin for 1 h, about 63.8% of the ENG microcrystal particles were deposited in the skin, thus achieving sustained intradermal administration for more than 1 week. In another study, the same team also reported a longer-acting ENG MN patch for 2 weeks’ delivery [79]. The PLGA needle-tips could be deposited *in situ* rapidly. The MNs were prepared by controllable casting-mould technique. ENG and PLGA were simultaneously dissolved in common solvent N-Methylpyrrolidine. And then the needle-tip layer was solidified at a low temperature to avoid the influence of high temperature melting on the stability of ENG. Meanwhile, the uniform crystallization of ENG in the needle-tip layer could be observed under a polarizing microscope.

It is particularly important to provide a new choice for female contraception, especially in developing countries. Prausnitz’s team surveyed women and suppliers in India and Nigeria on the acceptability of long-acting MN patches [42]. Women and suppliers were receptive to the creativity of long-acting contraceptive MN patches. After further discussion on the skin feeling of using MNs, they all eliminated their concerns about pain.

## Challenges in scale manufacture and regulations of polymeric MN systems

MN technology has great potential over traditional transdermal drug delivery and has been extended to clinical use by many researchers [122]. However, the popularization and application of MN technology is challenged by manufacturing barriers and unclear regulatory pathways [123, 124].

### Challenges in scale manufacture

In the preparation process of polymeric MNs, height, shape, spacing, penetration ability, and moisture all play important roles in the drug release profile and are key factors for design and development [13]. At present, whether polymeric MN technology can be promoted to industrial manufacturing relies upon industrial scalability, product quality control, and cost effectiveness.

Moulds are required to produce the desired shape, so the polymeric MN manufacturing processes can be divided into two categories: micro-moulding and lithography [125]. The micro-moulding method of fabrication requires an external force to fill the formula solution into the mould and is the most suitable for polymeric MNs. Generally, the filling mould methods mainly include centrifugation or vacuum [126]. Lithography, as a print process, transfers the master pattern of the MNs geometry to the surface of the substrate without any moulds [127]. The novel technique could use 3D printing and centrifugal lithography [125, 128]. 3D printing technology can significantly shorten the MNs product development cycle [129]. The advantages and disadvantages of polymeric MN manufacturing processes are summarized in Table 3.

**TABLE 3:** Overview of polymeric MN manufacturing processes.

Manufacturing process		Advantages	Disadvantages	Ref.
Micro-moulding	Centrifugation-filling	Cost-effective	Excess formula solution needs to be removed from the mould surface and wasted	[130, 131]
		Short centrifugal cycle	Risk of variation in each batch	
			More suitable for laboratory research	
	Vacuum-filling	Filling and drying steps can be combined	Excess formula solution also needs to be removed from the mould surface and disposed of	[132, 133]
		Cost-effective	Drug loading capacity	
		Easy to scale batch output		
Lithography	3D printing	Reduce waste and rapid prototyping	Layer-by-layer printing may affect mechanical strength	[134, 135]
		Very precise geometries	High-resolution instruments are expensive	
	Centrifugal lithography	Reproducibly produced	Available materials and API are limited	[136, 137]
		Suitable for fabricating MNs loaded with fragile biological drugs	Not ideal for industrial production	

To date, there is no record of biodegradable polymeric MNs on the global markets. Under GMP management, both standardized operating procedures and production process control need to be established to achieve repeatability for multiple batches, which would facilitate the industrial production of polymeric MNs.

### Challenges in regulations

In 2019, the PATCH organization formed the Microarray Patch Centre of Excellence, which aimed to mobilize efforts to accelerate MN development [123]. The current clearance procedure of MNs is for each individual application, and there is no perfect regulation construction for specific MN systems, which severely restricts the marketization of MN medical products [129].

The use of excipients often lacks rigorous safety profiles and data support, especially for intradermal use [138, 139]. Although the biocompatibility of MNs is high at present, some chemicals in MNs may accumulate in the skin. These polymer deposits may cause adverse effects on the skin or cause granulomas or local redness [7, 140]. The effects of long-term use of MNs on the skin need to be further discussed.

Further preclinical studies should be conducted to determine the pharmacokinetics, pharmacodynamics, and biosafety *in vivo* [141]. Early advice and planning by regulatory authorities are necessary. If the shortcomings of previous work and research are found in the later clinical stage, it will cause a very serious delay in clinical progress. Health and supervision departments should guide production industries to establish production process procedures and product quality standards suitable for this dosage form in accordance with Good Manufacturing Practice (GMP), to ensure the quality, consistency, and repeatability of the product [129]. Regulatory agencies require that large-scale production must be strictly sterile to prevent the occurrence of infection, which puts forward higher requirements for large-scale production manufacturers [91, 139] Finally, on the user’s view, it is necessary to establish the description of the administration method or the standard medication device to ensure the consistency of the dosage. This will accelerate effective clinical translation after product development.

## Discussion

Long-acting polymeric MN drug delivery systems, as a brand-new delivery platform, combines MN patches with sustained drug release technology. It has increased the potential of self-administration for patients with chronic diseases, improved patient compliance, and reduced the burden of fatigue of daily pills and the pain of injection. It could also avoid potential systemic toxicity when the drug is continuously released at a certain therapeutic concentration.

At present, in the global medical beauty industry market, there have been many anti-wrinkle and freckle MN products. The US Food and Drug Administration (FDA) published the regulatory consideration for microneedling products in 2020. It emphasized that the MNs in cosmetic belong to class II medical devices. In the pharmaceutical industry, the first NDA application to FDA was the Zolmitriptan coating-MNs developed by Zosano Pharma Corporation (United States). Several vaccine MNs (e.g., influenza and hepatitis B virus) have entered Phase I clinical studies. At present, several studies on doxorubicin delivery by dissolving MNs for the treatment of skin cancer have entered the stage of clinical trials (NCT04928222, NCT02192021, NCT03646188, and NCT03646188). However, unlike traditional dissolving or coating MNs, no long-acting polymeric MNs have entered the clinical stage. Despite many studies advancing long-acting MN-assisted drug therapy, there are still many issues worth considering in terms of large-scale commercial manufacturing, regulatory requirements, and patient acceptance. Long-acting polymeric MNs are a relatively new technology, and most of the current research has focused on the structure and application of MNs. They have shown effective therapeutic effects and significant superiority, giving us confidence and motivation. With more advanced preclinical and clinical studies, the future of MNs in long-acting drug delivery is promising.
