# Corrigendum: Recent progress of polymeric microneedle-assisted long-acting transdermal drug delivery

**DOI:** 10.3389/jpps.2025.14083

**Published:** 2025-01-27

**Authors:** Fanda Meng, Xinyu Qiao, Chenglong Xin, Xiaoli Ju, Meilin He

**Affiliations:** ^1^ College of Clinical and Basic Medicine, Shandong First Medical University & Shandong Academy of Medical Sciences, Jinan, China; ^2^ Shandong Center for Disease Control and Prevention, Jinan, China; ^3^ Yantai Key Laboratory of Nanomedicine and Advanced Preparations, Yantai Institute of Materia Medica, Yantai, Shandong, China

**Keywords:** polymeric, microneedles, drug delivery systems, transdermal, long-acting drug release

In the published article, there was an error in the **Funding** statement. The correct number for the Natural Science Foundation of Shandong Province, China is ZR2022QB149. The original text was: “This research was funded by the Natural Science Foundation of Shandong Province, China, grant number ZR2022QB14, and the Education and Teaching Reform Research Project of Shandong First Medical University and Shandong Academy of Medical Sciences, China, grant number XM2023016.” The correct Funding statement appears below.

